# Intracellular Transposition of Mobile Genetic Elements Associated with the Colistin Resistance Gene *mcr-1*

**DOI:** 10.1128/spectrum.03278-22

**Published:** 2022-12-13

**Authors:** Richard N. Goodman, Supathep Tansirichaiya, Michael S. M. Brouwer, Adam P. Roberts

**Affiliations:** a Department of Tropical Disease Biology, Liverpool School of Tropical Medicine, Liverpool, United Kingdom; b Department of Microbiology, Faculty of Medicine Siriraj Hospital, Mahidol University, Bangkok, Thailand; c Wageningen Bioveterinary Research, Lelystad, The Netherlands; University at Albany, State University of New York

**Keywords:** entrapment vector, insertion sequence, target site, antimicrobial resistance, plasmid, IS*Apl1*, Tn*7511*

## Abstract

Mobile colistin resistance (*mcr*) genes are often located on conjugative plasmids, where their association with insertion sequences enables intercellular and intracellular dissemination throughout bacterial replicons and populations. Multiple *mcr* genes have been discovered in every habitable continent, in many bacterial species, on both plasmids and integrated into the chromosome. Previously, we showed the intercellular transfer of *mcr-1* on an IncI1 plasmid, pMCR-E2899, between strains of Escherichia coli. Characterizing the intracellular dynamics of *mcr-1* transposition and recombination would further our understanding of how these important genes move through bacterial populations and whether interventions can be put in place to stop their spread. In this study, we aimed to characterize transfer events from the *mcr-1*-containing transposon Tn*7511* (IS*Apl1-mcr-1-pap2-*IS*Apl1*), located on plasmid pMCR-E2899, using the pBACpAK entrapment vector. Following the transformation of pBACpAK into our DH5α-Azi^r^/pMCR-E2899 transconjugant, we captured IS*Apl1* in pBACpAK multiple times and, for the first time, observed the IS*Apl1*-mediated transfer of the *mcr-1* transposon (Tn*7511*) into the chromosome of E. coli DH5α. Whole-genome sequencing allowed us to determine consensus insertion sites of IS*Apl1* and Tn*7511* in this strain, and comparison of these sites allowed us to explain the transposition events observed. These observations reveal the consequences of IS*Apl1* transposition within and between multiple replicons of the same cell and show *mcr-1* transposition within the cell as part of the novel transposon Tn*7511*.

**IMPORTANCE** By analyzing the intracellular transfer of clinically relevant transposons, we can understand the dissemination and evolution of drug resistance conferring mobile genetic elements (MGEs) once a plasmid enters a cell following conjugation. This knowledge will help further our understanding of how these important genes move through bacterial populations. Utilizing the pBACpAK entrapment vector has allowed us to determine the mobility of the novel *mcr-1*-containing transposon Tn*7511*.

## INTRODUCTION

Antimicrobial resistance (AMR) is a threat to human health globally, with recent studies estimating that it causes millions of deaths annually, making it a leading cause of death, along with HIV and malaria (1). Colistin (polymyxin E) is a key last-resort antibiotic which can be used to treat Gram-negative infections when other antimicrobial chemotherapeutics fail (2), but recently, mobile colistin resistance (*mcr*) genes have been found in hospital and community settings around the world. Originally, colistin resistance was thought to be solely chromosomally linked; however, in 2016, *mcr-1* was found on an IncI2 plasmid in Escherichia coli isolated from a pig in China (3). Since then, multiple *mcr* variants have been discovered on every continent except Antarctica, in a diverse set of bacterial species, including Escherichia coli, Klebsiella pneumoniae, Salmonella enterica, and Shigella sonnei, located on both plasmids and the chromosome (4).

Antimicrobial resistance genes disseminate among bacteria via mobile genetic elements (MGEs), which can transfer between bacterial communities both intercellularly, for example through conjugation, transformation, and transduction events, and intracellularly, through transposition and recombination events. Understanding the intracellular dynamics of MGEs associated with AMR genes within clinical and environmental isolates is a prerequisite to effective interventions aimed at reducing the spread of AMR.

Entrapment vectors are engineered plasmids which confer a phenotypic change to the host cell when an MGE is inserted into the backbone of the plasmid (5). Previously, the entrapment vector pBACpAK was engineered to capture MGEs in, and from, clinical isolates of E. coli in order to understand the intracellular dynamics of various MGEs (6, 7). pBACpAK utilizes a repressor-antibiotic resistance gene system to detect the insertion of MGEs. Along with a chloramphenicol resistance gene (*catA1*), pBACpAK contains a *cI* repressor upstream of a *tetA* gene; therefore, it does not normally confer tetracycline resistance. However, when a MGE is inserted into the *cI* repressor, the repression of *tetA* is alleviated, and the replicon confers tetracycline resistance to the host cell (8). This enables positive selection for transposition using antibiotic-supplemented agar. pBACpAK has already been shown to capture translocatable units (TUs) by transforming pBACpAK into clinical E. coli isolates (7) and has been shown to capture an entire NDM-1 containing 119-kb plasmid by transferring conjugative plasmids into an E. coli strain containing pBACpAK which has been cured of all chromosomal MGEs (6).

We used pBACpAK to capture MGEs from the conjugative plasmid pMCR-E2899, which contains a copy of *mcr-1* and was previously shown to transfer intercellularly between E. coli isolates (9). Following the successful transfer of pBACpAK into E. coli DH5α-Azi^r^/pMCR-E2899 and screening for tetracycline resistance in the transformants, we observed multiple transposition events from plasmid pMCR-E2899 into pBACpAK and multiple IS*Apl1*-mediated transfer events into the E. coli DH5α chromosome.

## RESULTS

### Assessing resistance of the recipient strain and plasmid pBACpAK.

Based on a bioinformatic analysis of the recipient strain DH5α-Azi^r^/pMCR-E2899, no resistance genes were found for either chloramphenicol or tetracycline. This was confirmed *in vitro* by plating the strain onto LB agar with chloramphenicol and tetracycline: no growth was observed, which confirmed the suitability of this strain for the pBACpAK transformation and subsequent transposition assay.

### Transfer of plasmid pBACpAK into E. coli DH5α-Azi^r^/pMCR-E2899.

Following the transformation of pBACpAK into E. coli DH5α-Azi^r^/pMCR-E2899, colonies were subcultured, and 8 colonies were selected for PCR. The *cI*-*tetA* and *mcr-1* gene fragments were amplified from 2 of 8 and 7 of 8 colonies, respectively. Amplification of the *cI-tetA* region is diagnostic for the presence of pBACpAK; amplification of *mcr-1* is diagnostic for the presence of pMCR-E2899. Four of the eight colonies were taken forward; all had an amplicon for *mcr-1*, and two had amplicons for *cI-tetA*. At this stage, the lack of an amplicon for *cI-tetA* may be indicative either of an insertion within the *cI-tetA* region disrupting the target for the primers or the lack of pBACpAK within the cell.

### Screening for transposants with insertion of MGEs into pBACpAK.

**FIG 1:**
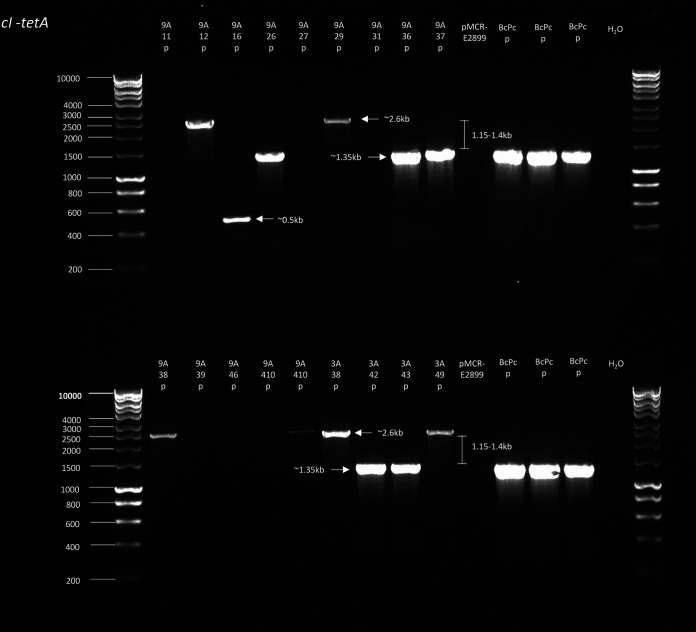
*cI-tetA* amplification of plasmid DNA isolated from DH5α-Azi^r^/pMCR-E2899/pBACpAK colonies growing on chloramphenicol, colistin, and tetracycline. Plasmid DNA was used as the template; it was extracted using the NEB Monarch plasmid miniprep kit. BcPc, plasmid pBACpAK; P, plasmid extraction.

**FIG 2:**
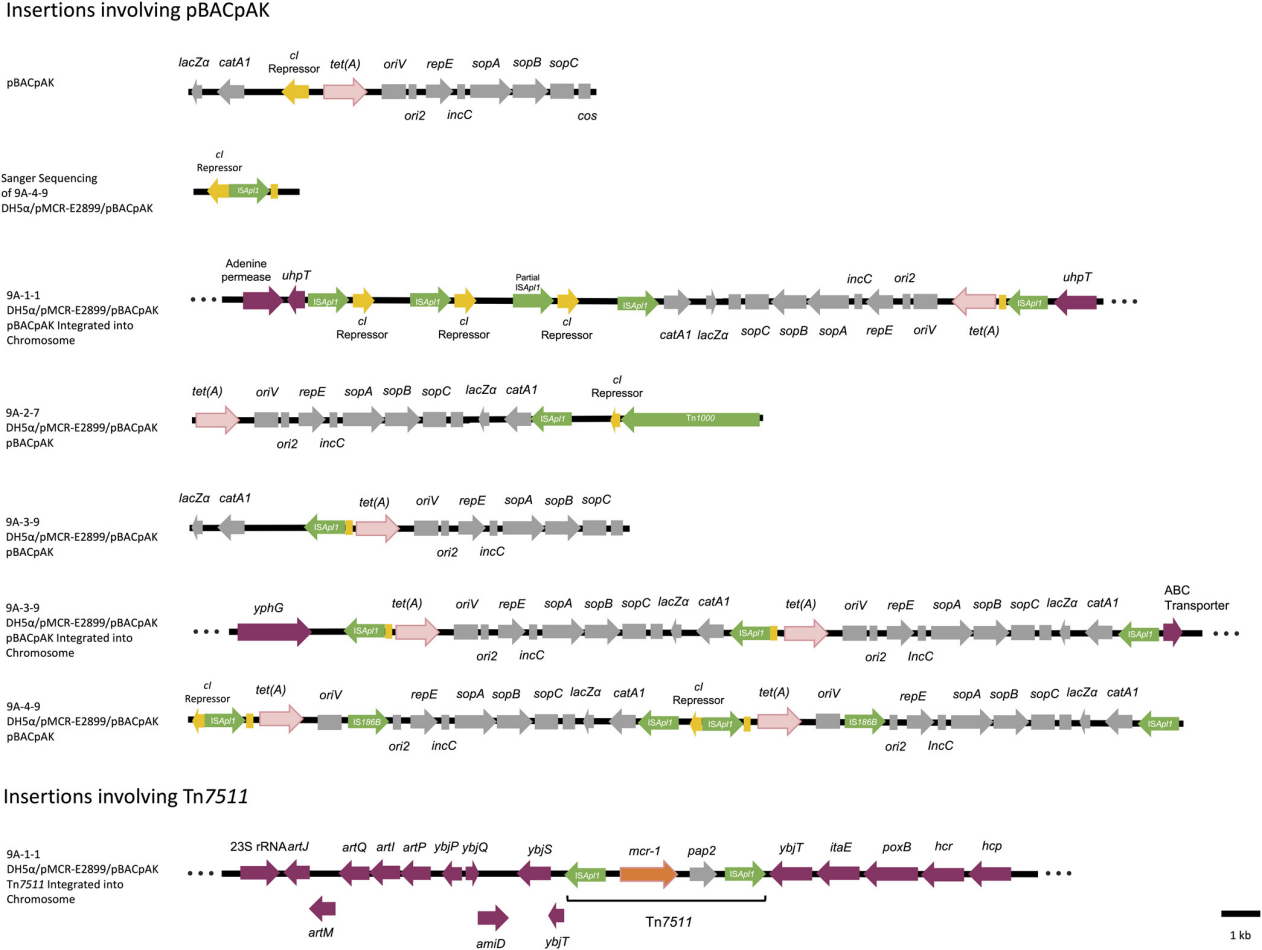
Structures of the pBACpAK plasmids capturing MGEs and structures of pBACpAK and Tn*7511* integrating into the chromosome. The colors of the boxes represent different genetic features: red, *tetA*; yellow, *cI* repressor; green, MGEs; gray, pBACpAK genes; purple, chromosomal genes. The three dots at the terminals of the sequences represent integration into the chromosome.

Colonies were screened for transposition by PCR amplification, which showed several clones with a 1.15- to 1.4-kb increase in the size of the *cI-tetA* amplicon (Fig. 1). Sanger sequencing of the *cI*-*tetA* region confirmed insertions of IS*Apl1* in the *cI* region (Fig. 2).

### Genomic analysis of whole-genome sequences of selected transposants.

Isolates for which no amplicon was seen in the MyTaq PCR amplification of the *cI-tetA* region were sequenced using both long- and short-read sequencing technologies. Table 1 shows the sequences assembled into contigs, each relating to the separate replicons (both chromosome and plasmids) within the cell, along with any insertions observed. Four independent transposants were sequenced: 9A-1-1, 9A-2-7, 9A-3-9, and 9A-4-9 (see Table 2 for information on how these transposants were selected and named). As each colony was confirmed to be DH5α-Azi^r^/pMCR-E2899/pBΑCpAK prior to sequencing, it was expected that each would contain three replicons: the DH5α chromosome (4,585,426 bp), plasmid pBACpAK (10,986 bp), and plasmid pMCR-E2899 (107,399 bp).

**TABLE 1:** Insertions observed from whole-genome sequencing of 4 transposants (DH5α-Azi^r^/pMCR-E2899/pBACpAK colonies)

Colony/name	Replicon/contig no.^*a*^	Replicon/contig name	Replicon/contig size (bp)	Observed insertions^*b*^		
				Plasmid/transposon	AMR gene(s)	Insertion sequence(s)
9A-1-1	1	Chromosome	4,615,780	Tn*7511*	*mcr-1*	IS*Apl1* (×11)
				pBACpAK	*tetA*, *catA1*	
	2	pMCR-E2899	107,891			Partial IS*Apl1*
9A-2-7	1	Chromosome	4,586,787			IS*Apl1* (×2)
	2	pBACpAK	18,037	Tn*1000*		IS*Apl1*
	3	pMCR-E2899	107,399			
9A-3-9	1	Chromosome	4,613,846	pBACpAK (×2)	*tetA* (×2), *catA1* (×2)	IS*Apl1* (×10)
	2	pBACpAK	10,343			IS*Apl1*
	3	pMCR-E2899	108,470			IS*Apl1*
9A-4-9	1	Chromosome	4,585,712			IS*Apl1*
	2	pBACpAK (duplicated)	28,952		*tetA*, *catA1*	IS*Apl1* (×4), IS*186B* (×2)
	3	pMCR-E2899	107,399			

*^a^*As each contig is a replicon here, the two words are used interchangeably.

b Numbers in parentheses signify the number of insertions of that particular genetic feature within the replicon.

**TABLE 2:** DH5α-Azi^r^/pMCR-E2899/pBACpAK transformants observed to grow on colistin, chloramphenicol and tetracycline (transposants)^*a*^

Assay name (method)	Colony name	No. of colonies:			No. of colony PCR amplicons observed	
		Observed	Picked	Recovered	*cI-tetA*	*tetA*
3A (16 h broth, 42 h plate)	3A-3	15	10	10	0	9
	3A-4	17	10	10	0	8
9A (24 h broth, 42 h plate)	9A-1	125	10	10	1	10
	9A-2	122	10	10	0	8
	9A-3	16	10	9	0	8
	9A-4	144	10	10	3	8

a Colonies were observed in two separate assays, in which colonies confirmed to contain pBACpAK were incubated in LB plus colistin and chloramphenicol for up to 24 h and then aliquoted out onto LB plates at several time points. All transposants are the same strain: DH5α/pMCR-E2899/pBΑCpAK. The transposant names represent the assay name, transformant number, and transposant number. For example, 9A-1-1 refers to transposant colony number 1 selected from the transformant 9A-1 DH5α-Azi^r^/pMCR-E2899/pBACpAK.

In the transposants 9A-2-7, 9A-3-9, and 9A-4-9, the three expected replicons were detected, which allowed us to study the MGE insertions into pBACpAK. In the 9A-2-7 transposant, IS*Apl1* had transferred into the pBACpAK plasmid, just downstream of the CHL^r^ gene (*catA1*), and Tn*1000* was also found to have transferred into the *cI* gene of pBACpAK. Tn*1000* was present in the DH5α chromosome (see Fig. 2). In the 9A-3-9 transposant, there was an insertion of IS*Apl1* into the *cI* repressor gene of pBACpAK, upstream of the *tetA* gene. IS*Apl1* was transferred from the pMCR-E2899 plasmid in each case, as DH5α was shown to have no copies of IS*Apl1* within its chromosome. In the transposant 9A-4-9, two copies of IS*186B* and four copies of IS*Apl1* were inserted into pBACpAK (see Fig. 2). IS*186B* is present in the DH5α chromosome.

In 9A-1-1, unlike the other three transposants, there were only two replicons, due to the complete insertion of plasmid pBACpAK into the 9A-1-1 DH5α chromosome within the *uhpT* gene, between positions 4500607 and 4520319. In 9A-1-1, plasmid pBACpAK was flanked by IS*Apl1* in opposing orientations, as shown in Fig. 2. This makes the IS*Apl1*-flanked pBACpAK a composite transposon (Tn), according to previously published nomenclature (10). Upstream of the pBACpAK insertion, there were two more copies of IS*Apl1* and one partial IS*Apl1* sequence, which is likely due to recombination. In the transposant 9A-3-9, pBACpAK was also found to be inserted into the DH5α chromosome, like in 9A-1-1; however, there were two inserted tandem pBACpAK copies (see Fig. 2), again presumably mediated by IS*Apl1*. The IS*Apl1* sequences flanked the duplicated pBACpAK in direct orientation, again technically forming a composite transposon carrying two *tetA* genes and two *catA1* genes. In the transposant 9A-4-9, while there were no chromosomal insertions detected, a duplicated pBACpAK episome was found and shown to have a size of 28,952 bp (Fig. 2). This duplicated pBACpAK was aligned against the inserted duplicate pBACpAK in the chromosome of 9A-3-9, and there was consensus in 27,208 of the 28,952 bp, with only 2 mismatches and 6 gaps. It is therefore possible that the plasmid in 9A-4-9 shows the structure prior to insertion into the DH5α chromosome.

The entire *mcr-1*-containing transposon (IS*Apl1-mcr-1-pap2-*IS*Apl1*) from pMCR-E2899 was found to be inserted into the 9A-1-1 DH5α chromosome at a separate site to the pBACpAK insertion point (position 1506096), within the *ybjT* gene. Since we observed the transposition of this transposon within this study and because it has a novel architecture in comparison to Tn*6330* (see Fig. S2 in the supplemental material), it was designated Tn*7511* by the Transposon Registry (11). Within Tn*7511* on plasmid pMCR-E2899 of 9A-1-1, a partial IS*Apl1* of 492 bp was observed (see Table 1); this is possibly a result of recombination. Since the Tn*7511* copy that transposed into the 9A-1-1 chromosome did not have this additional feature, it likely happened post-Tn*7511* transposition. This genomic analysis allows a picture to be built of the intracellular transfer pathways, as each colony acts as a snapshot of the intracellular movements involved between the three replicons present in the cell.

### Insertion site motifs for IS*Apl1* and Tn*7511*.

**FIG 3:**
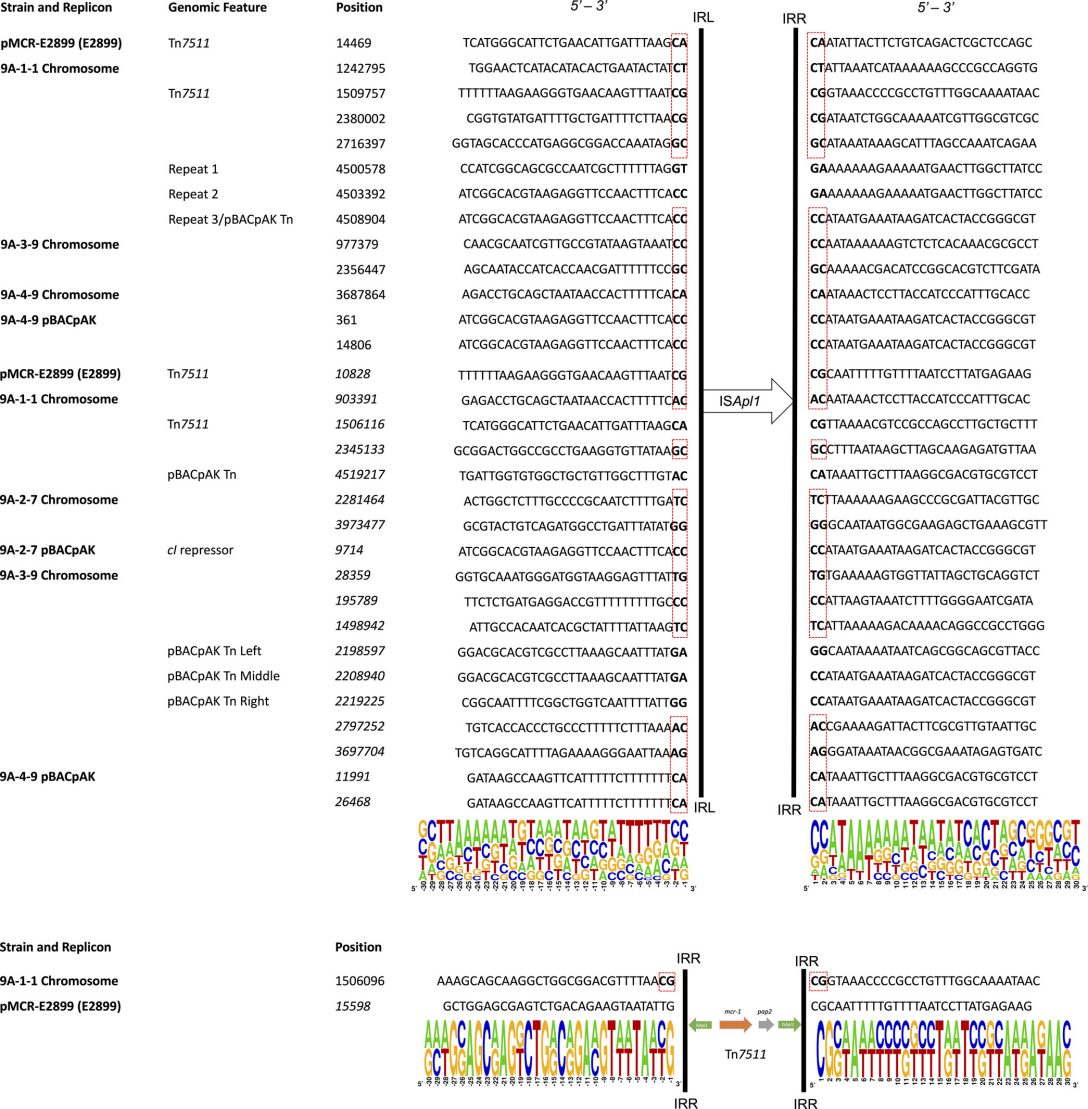
Complete sequences of IS*Apl1* and Tn*7511* insertion sites. For sequences of IS*Apl1* insertion sites (*n *= 31), the last 18 sequences were converted to their reverse complement prior to alignment, as IS*Apl1* was inserted with the opposite orientation. The coordinates of these reverse orientation insertions are denoted in italics. All sequences of Tn*7511* (*n *= 2) with the second sequence converted to their reverse complement prior to alignment, as Tn*7511* was inserted with the opposite orientation. Target site duplications (TSDs) are highlighted in a red box.

The insertion sites for both IS*Apl1* and Tn*7511* in the 4 transposants were analyzed. Fig. S1 shows the consensus motifs from IS*Apl1* insertions, separated by the orientation in which they were inserted. Eighteen insertions were oriented as 5′-IRR (right inverted repeat)-IS*Apl1*-IRL (left inverted repeat)-3′ and 13 as 5′-IRL-IS*Apl1*-IRR-3′, making 31 observable insertions in total. As shown in Fig. 3, the majority of insertions occurred in a central GC-rich tetramer within flanking AT-rich regions. The 2 bp upstream and downstream always contained at least one G or C, and in 45% of insertions, the central tetramer was made up of entirely C or G. The insertion sites were also flanked by 2-bp direct repeats in 77% of all IS*Apl1* insertions observed (24/31), as shown in Fig. 3. These sites are target site duplications (TSDs), which are generated upon the insertion of IS*Apl1* (12, 13). Tn*7511* was also found to be inserted into a GC-rich tetramer flanked by an AT-rich region (see Fig. 3), which is expected as IS*Apl1* flanks the Tn and likely mediates its transposition. Interestingly, the insertion site in the 9A-1-1 chromosome contained a TSD, but the original site in pMCR-E2899 did not (see Fig. 3).

### PCR to identify a putative (IS*Apl1*)_2_ circular intermediate.

**FIG 4:**
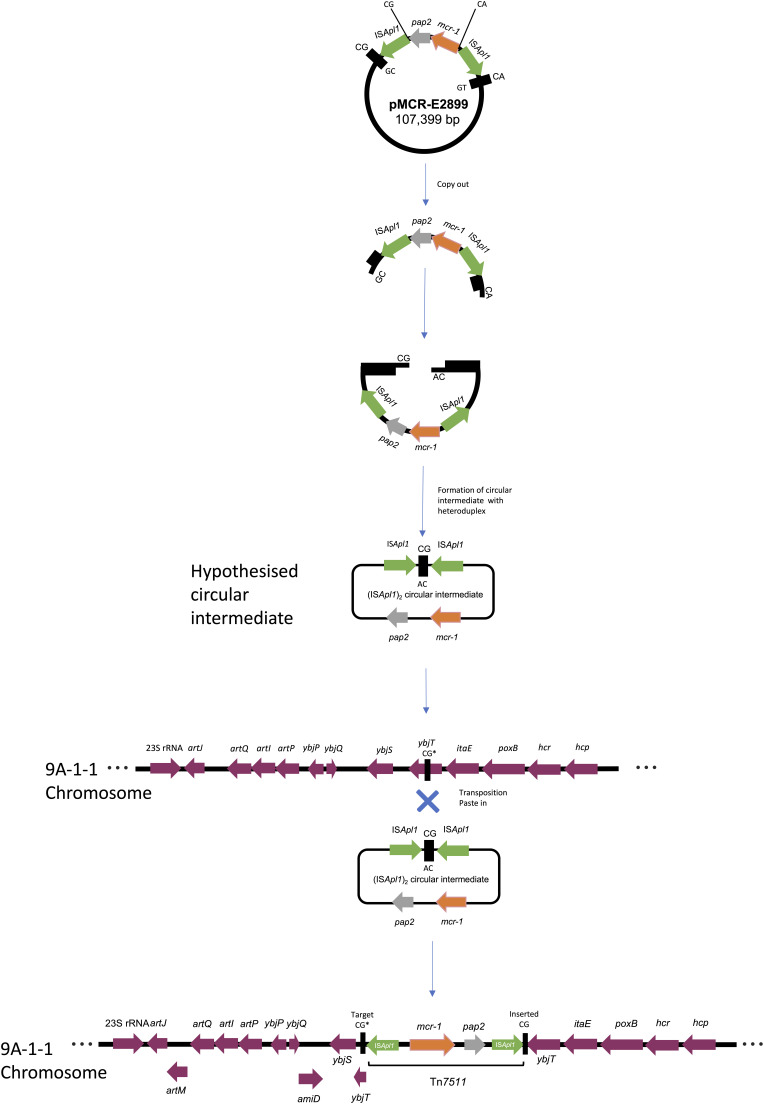
Genetic mechanism of Tn*7511* transposition from pMCR-E2899 to the 9A-1-1 DH5α E. coli chromosome via a circular intermediate.

To test the hypothesis that an (IS*Apl1*)_2_ circular intermediate was formed during the transposition of Tn*7511* from pMCR-E2899 to the DH5α chromosome in the transposant 9A-1-1 (see Fig. 4), we ran a PCR to amplify the predicted joint of a Tn*7511* circular intermediate using primers facing outwards from *mcr-1* (see Table S1). However, no amplicon was visible at the expected size of 3,180 bp (data not shown); the full double IS*Apl1*-containing circular intermediate would be 4,685 bp in size, including the 2-bp TSD.

## DISCUSSION

Entrapment vectors have been shown to capture MGEs in many species, including both Gram-positive and Gram-negative organisms. Since this method relies on the intracellular movement of MGEs within the cells, it does not require any prior sequencing information and has the potential to capture novel MGEs. In our study, we demonstrated multiple insertions of IS*Apl1* from the *mcr-1*-containing plasmid pMCR-E2899 into the *cI* repressor region of pBACpAK and into other regions of pBACpAK and the DH5α chromosome. The originally transferred plasmid, pMCR-E2899, contains only two copies of IS*Apl1*, which flank *mcr-1*, but it does also contain multiple copies of IS*1* and copies of IS*26* and IS*Sso4*; however, we only detected transfer of IS*Apl1* in our assays, whereas previously, we had also detected the transposition of IS*26* into pBACpAK (6). We did also detect Tn*1000* and IS*186B* from the host cell chromosome into pBACpAK, illustrating the multidirectional nature of between-replicon MGE transfer upon the acquisition of a new plasmid by a host cell.

Using whole-genome sequencing (WGS), the IS*Apl1*-mediated duplication and insertion of pBACpAK into the DH5α chromosome was observed, and in the case of one sequenced transposant (9A-1-1), both pBACpAK and the *mcr-1* containing transposon, Tn*7511*, were inserted into the DH5α chromosome, allowing the cell to acquire three chromosomally integrated resistance genes, *mcr-1*, *catA1*, and *tetA*. We found the insertion of pBACpAK into the chromosome in differing conformations (Fig. 2). In the transposant 9A-1-1, we observed the amplification of the interrupted *cI* repressor within pBACpAK, itself within the chromosome of E. coli DH5α and likely catalyzed by IS*Apl1* (Fig. 2). Insertion sequence (IS)-mediated amplification of genes can have clinically important consequences. Resistance gene amplification, for example, can lead to heteroresistance, where a subset of cells in a clonal bacterial population display temporary phenotypic resistance, and a subset display phenotypic susceptibility (14, 15). Recent examples of this mechanism have included the amplification of *bla*_TEM-1B_ (16), *tetA*, *aac(3)-IId*, and *sul1* (17). It is unclear what selective pressure would lead to selection of the amplification of an interrupted *cI* repressor, and it may be that we are seeing a snapshot of a background level of IS*Apl1*-mediated amplification.

IS*Apl1* was initially found inserted into the *cI* repressor by using PCR and Sanger sequencing; it is an IS*30* family insertion sequence originally from Actinobacillus pleuropneumoniae (13), and it flanks the *mcr-1*-containing transposons Tn*7511* (this study), Tn*6390* (18), and Tn*6330* (19), of which Tn*6330* is the most well characterized. Tn*7511* is similar to Tn*6330* and Tn*6390*, except that the flanking IS*Apl1* copies are in different orientations (see Fig. S2 in the supplemental material). Tn*6330* has two copies of IS*Apl1* in direct orientation: IRL-IS*Apl1-mcr-1-pap2-*IS*Apl1-*IRR (20). Tn*6390* has both copies of IS*Apl1* orientated inwards: IRL-IS*Apl1-mcr-1-pap2-*IS*Apl1-*IRL (18). Finally, Tn*7511*, from this study, has both copies of IS*Apl1* orientated outwards: IRR-IS*Apl1-mcr-1-pap2-*IS*Apl1-*IRR. It has already been shown that the presence of two flanking IS*Apl1* sequences in Tn*6330* indicates that the transposon is mobile and primed for intracellular transfer (21). Losing either of the flanking IS*Apl1* sequences from the transposon will decrease the chance of transfer, while increasing plasmid stability (22). Tn*6330* has been shown to be stable with and without exposure to colistin over 30 generations (23), suggesting that once integrated, *mcr-1* is not readily lost, even after the lifting of colistin exposure. IS*Apl1* is a highly active insertion sequence that has been shown in this study and others (24) to move independently of *mcr-1*.

Here, we have shown the transfer of the entire colistin resistance-encoding transposon Tn*7511*; this transfer was observed to occur from plasmid pMCR-E2899 into the DH5α chromosome. The transfer of other *mcr-1* transposons such as Tn*6330* has previously been characterized through analyzing genomic data sets (21) but insertion has never before been shown without being differentially marked with another antibiotic resistance gene (25).

WGS also revealed the details of multiple IS*Apl1* and Tn*7511* insertion sites, as shown in Fig. 3. The advantage of the entrapment system used in this study is that the entire intracellular system is unchanged between insertion sites. IS*Apl1* was found to be inserted into a central GC-rich tetramer within a distal AT-rich region, in agreement with previous work reporting IS*Apl1* insertions across multiple genomes in sequence databases (12, 19, 24–26). IS*30* family transposases, of which IS*Apl1* is a member, have been found to target imperfect palindrome sequences with a central region of GC bias and a distal region rich in AT (27).

The IS*Apl1* insertion sites reported here were flanked by direct repeats in 77% of cases (see Fig. 3), forming target site duplications (TSDs), as reported previously in IS*Apl1* insertion sites (12). The movement of IS*Apl1* involves a circular intermediate, which contains 2 bp of host DNA at the joint of the circular intermediate, between the ends of the transposon, and insertion into a new target site adds the integrated 2 bp to the 2 bp of the host DNA to create the TSD (13, 27, 28). The presence of TSDs is due to direct insertions by this copy-out and paste-in transposition mechanism shared by members of the IS*30* family (12). In the 33% of insertions without TSDs, it is likely that the mechanism is the result of either recombination, as in the case of the tandem repeats upstream of the inserted pBACpAK sequence in the 9A-1-1 chromosome, or transposition of a larger IS*Apl1*-flanked transposon, as in the case of the IS*Apl1* sequence on the right side of the inserted pBACpAK sequence and the IS*Apl1* sequence on the right side of Tn*7511* in the 9A-1-1 chromosome (see Fig. 2). All other IS*Apl1* insertion sites in the 9A-1-1 chromosome contain TSDs except these. Interestingly, the inserted pBACpAK sequence in transposant 9A-3-9 also lacks flanking TSDs in any of the three IS*Apl1* sequences within it. This suggests that in both transposants where the formation of an integrated pBACpAK sequence was observed, dual mechanisms of recombination and transposition led to the integration of pBACpAK into the chromosome. In other studies, the lack of TSDs was due to a deletion (12), which could be an alternative explanation. However, this is unlikely in our case, as all IS*Apl1* sequences that lack flanking TSDs can be accounted for by either recombination or transposition.

This analysis also revealed that the copy of Tn*7511* which was inserted into the 9A-1-1 chromosome was flanked by TSDs, in contrast to the copy of Tn*7511* in pMCR-E2899, which did not have flanking TSDs (see Fig. 3). This suggests that Tn*7511* moved from pMCR-E2899 into the chromosome by a copy-out–paste-in mechanism of transposition the same as that of Tn*6330* (19, 26). This mechanism involves a circular intermediate containing 2 bp of host DNA between the two ligated copies of IS*Apl1*, which, upon insertion, introduced a signature TSD, in this case a CG (see Fig. 4). However, when we ran a PCR to amplify the predicted Tn*7511* (IS*Apl1*)_2_ circular intermediate, no amplicon was visible at the expected size of 3,180 bp. This lack of amplification may be due to the fact that the transposition event could be rare and the circular intermediate nonreplicative. Further to this, another hypothesis was suggested by a reviewer to explain why this transposition is so rare and why it was only observed once in our study. It is possible that a single mutation occurred in the 3′-end TSD of the IS*Apl1* sequence upstream of *mcr-1*, changing the CA to a CG (see Fig. 4 for reference). If this was the case, a circular intermediate would be formed, adjoined by a TSD of CG, which would then allow the transposition of the Tn*7511* transposon into the chromosome of 9A-1-1 at the CG target site. Comparative genomics of the insertion site in 9A-1-1 with the same site in the other transposants showed that Tn*7511* was inserted into the *ybjT* gene (see Fig. S3); this encodes a putative NAD(P)-dependent oxidoreductase which makes up a collection of genes responsible for stress-induced mutagenesis in E. coli.

The lack of TSDs flanking Tn*7511* in pMCR-E2899 suggests that the acquisition of Tn*7511* in pMCR-E2899 did not occur via copy-out–paste-in transposition but possibly by the acquisition of Tn*7511* on a larger mobile genetic element or through a multistep process of IS*Apl1* transposition, resulting in the formation of Tn*7511* within pMCR-E2899. The latter is the case for Tn*6330* (19), where the internal ends of the flanking IS*Apl1* copies (AT in the upstream copy and CG in the downstream copy) are conserved, which likely constitutes the TSDs from the original formation of Tn*6330*, i.e., AT-IS*Apl1-*AT*-mcr-1-pap2-*CG-IS*Apl1-*CG, with two IS*Apl1* copies transposed on either side of the conserved *mcr-1-pap2* sequence (19). In the Tn*7511* sequence found in pMCR-E2899, the upstream copy of IS*Apl1* was flanked by an CG dimer TSD and the downstream copy by a CA dimer TSD, i.e., CG-IS*Apl1*-CG-*pap2*-*mcr-1*-CA-IS*Apl1*-CA (see Fig. 4), which suggests that Tn*7511* did not integrate into pMCR-E2899 as an individual transposable element.

Several possible pathways could have resulted in the formation of Tn*7511*. The only explanation that is not possible is copy-out–paste-in transposition, as a TSD does not flank the Tn*7511* sequence in pMCR-E2899. This analysis of the genetic context of the insertion sites, especially the presence or absence of TSDs, gives greater insight into the mechanism of transfer of both IS*Apl1* and Tn*7511* within the cell and the movement and formation of known and novel transposons.

The comparison of TSDs at transposon insertion sites also carries limitations in that the TSDs are only 2 bp long, so it is difficult to confirm whether the TSDs are genuine or if the duplicated sequence is serendipitous. However, in this study we compared 31 insertion sites in the same intracellular system, so the analysis was able to confirm the TSDs and the mechanism of transposition for Tn*7511*.

Although in this study WGS showed the insertion of both Tn*7511* and pBACpAK into the chromosome of DH5α, the assay is limited in the fact that isolates will only be selected if there is an insertion in the *cI* repressor region of pBACpAK. Therefore, it is only in situations of extensive intracellular transfer that insertion into both the *cI-tetA* region of pBACpAK and the chromosome can be observed. With the declining cost and increasing ease of sequencing (using both long and short reads), using WGS to analyze all isolates resistant to tetracycline is becoming a more realistic option, as is the use of deep sequencing on populations of cells to obtain an aggregate insertional map.

In conclusion, we have characterized multiple MGE transpositions within, and between, a 3-replicon system (DH5α chromosome, pMCR-E2899 containing *mcr-1*-encoding Tn*7511*, and pBACpAK), detecting transfer between sites in the same plasmid and transposition between plasmids, from plasmids to the chromosome, and from the chromosome to a plasmid. We observed IS*Apl1*-mediated MGE duplication, gene amplification, and integration of pBACpAK into the chromosome. We also detected the IS*Apl1*-mediated transposition of the *mcr-1*-containing novel transposon Tn*7511* into the chromosome of E. coli DH5α, providing insight into how this clinically important transposon moves intracellularly.

## MATERIALS AND METHODS

### Bacterial strains, plasmids, and culture conditions.

In this study, strain E. coli DH5α-Azi^r^/pMCR-E2899 (9) was used as the recipient in transformation assays. The complete genome of E. coli DH5α-Azi^r^/pMCR-E2899 was screened for tetracycline and chloramphenicol resistance genes using ResFinder (29), and the strain was plated on a medium containing both chloramphenicol and tetracycline to detect phenotypic resistance. Bacteria were grown in lysogeny broth (LB) medium supplemented with antibiotics (Sigma-Aldrich, UK) at the following concentrations: 2 μg/mL colistin, 12.5 μg/mL chloramphenicol, and 5 μg/mL tetracycline. The entrapment vector pBACpAK was used as the transforming plasmid. pBACpAK has been described previously to capture mobile genetic elements within E. coli (7).

### Preparation of DH5α-Azi^r^/pMCR-E2899 electrocompetent cells.

E. coli DH5α-Azi^r^/pMCR-E2899 cells (grown in 2 μg/mL colistin) were made electrocompetent following a previously described protocol (30). After 4 glycerol washes, the bacterial cell pellet was resuspended in 100 μL of 10% glycerol and stored at −80°C in aliquots of 50 μL. All centrifugation steps were carried out in a 5810R centrifuge (Eppendorf, Germany).

### Extraction of plasmid pBACpAK from E. coli MDS/pBACpAK.

The pBACpAK plasmid DNA was extracted from strain E. coli MDS/pBACpAK (6) using the Monarch plasmid miniprep kit according to the manufacturer’s instructions (New England Biolabs, USA), except that 7.5 mL of bacterial cell culture grown for 18 h at 37°C in colistin (2 μg/mL) was used for the protocol, and during the lysis stage, there was a 5-min incubation in buffer B2. The DNA was eluted in 60 μL of elution buffer.

### Transformation of pBACpAK into DH5α-Azi^r^/pMCR-E2899 recipient cells.

The transformation was carried out by electroporation, for which 50 μL of competent cells were transferred to a prechilled 1.5-mL microcentrifuge tube and mixed with 10 to 100 ng of pBACpAK plasmid DNA. This was transferred to a prechilled 0.1-cm electroporation cuvette (Bio-Rad, Watford, UK), and electroporation was carried out using a MicroPulser apparatus (Bio-Rad) under the conditions appropriate for E. coli and the cuvette gap (1.8 V, 200 Ω, and 25 μF). Immediately, 950 μL of Super Optimal broth with catabolite repression (SOC) medium (New England Biolabs, USA) was added. The SOC cell suspension was transferred to a 50-mL Falcon tube and incubated at 37°C and 200 rpm for 1 h, 2 h, and 3 h. A 100-μL aliquot of cell culture at different time points was then plated onto LB agar supplemented with colistin and chloramphenicol and incubated at 37°C for 18 to 24 h.

### Screening for transformants.

The presence of pBACpAK was confirmed by carrying out a colony PCR with the *cI-tetA* primers (see Table S1 in the supplemental material) and 2× MyTaq Redmix (Bioline, Meridian Biosciences, OH, USA). The PCR conditions were as follows: initial denaturation at 95°C for 1 min, followed by 35 cycles of denaturation at 95°C for 15 s, annealing at a primer-specific temperature for 15 s, and extension at 72°C for 1 min for every 1 kb of amplicon. The samples were then cooled to 4°C. The PCR products were visualized by gel electrophoresis; this involved separation on 1% (wt/vol) agarose using GelRed (Biotium, Cambridge, UK) at a concentration of 0.1 μg/mL.

### Screening for transformants with insertion of MGEs into pBACpAK (transposants).

Successful transformants (subsequently referred to as DH5α-Azi^r^/pMCR-E2899/pBACpAK) were transferred to LB broth with colistin and chloramphenicol and incubated for either 4, 16, 20, or 24 h at 37°C and 200 rpm. At each time point, 100-μL aliquots of neat and dilutions of 10^−4^ and 10^−6^ were plated onto either LB with chloramphenicol plus tetracycline, LB with chloramphenicol plus tetracycline plus colistin, or LB only. These plates were incubated at 37°C for 42 h, and when observed, colonies were subcultured on fresh LB chloramphenicol plus tetracycline plus colistin plates. Ten colonies were taken from each plate (see Table 2), and colony PCR was performed to amplify both the *cI-tetA* region and the *tetA* region (see Table S1). PCRs used 2× MyTaq Redmix (Bioline, Meridian Biosciences), and conditions were as described above.

### PCR screening of transposant genomic and plasmid DNA.

Plasmid DNA was extracted from subcultured transformants using the Monarch plasmid miniprep kit according to the manufacturer’s instructions (New England Biolabs), with the modifications described above. Bacterial cell culture (7.5 mL) was used for the extraction, after being supplemented with chloramphenicol, colistin, and tetracycline and grown at 37°C and 200 rpm for 42 h. The plasmid DNA was eluted in 60 μL elution buffer. Genomic DNA (gDNA) was extracted from subcultured transformants using the Monarch genome DNA purification kit (New England Biolabs) according to the manufacturer’s instructions. Overnight bacterial cell culture (1 mL) was used in the extraction. DNA was eluted with 100 μL gDNA elution buffer. PCR amplification of the *cI-tetA* region was repeated using this extracted plasmid and genomic DNA. MyTaq Redmix (2×; Bioline, Meridian Biosciences) was used, with the extension time increased to 5 min, in order to amplify the *cI* region and any inserts. All PCR products were visualized by gel electrophoresis as described above.

### Sanger sequencing of transposants.

Where an amplicon was larger than the wild-type pBACpAK *cI* region, it was sent for Sanger sequencing. The PCR products were purified using the Monarch PCR and DNA cleanup kit (New England Biolabs), according to the manufacturer’s protocol. The DNA was eluted with 10 μL of elution buffer. The purified PCR products (5 μL) were mixed with 5 μL of the *cI-tetA* primer and sent for Sanger sequencing (Genewiz, Azenta Life Sciences, Germany). Chromatograms were analyzed for quality and clear base distinction. Primers were designed iteratively to primer walk along the unknown insert region within the *cI*-repressor; these are all shown in Table S1.

### Whole-genome sequencing of transposants.

Two transposant isolates (9A-1-1 and 9A-3-9; see Table 2) were sent to MicrobesNG (Birmingham, UK) for short-read sequencing using the HiSeq X10 platform (Illumina, USA). The bacterial isolates were prepared according to the MicrobesNG strain submission procedures. The data were analyzed by MicrobesNG with a simple bioinformatics workflow, including quality filtering and sequence read trimming.

Two samples (9A-2-7 and 9A-4-9) were sequenced in our laboratory using the iSeq 100 sequencing system (Illumina). Genomic DNA was extracted using a Fire Monkey high-molecular-weight (HMW) DNA extraction kit (RevoluGen, UK). Library prep was carried out using the NEBNext Ultra II FS DNA library prep with sample purification beads (New England Biolabs) according to the manufacturer’s protocol for the FS DNA library prep kit (E6177) with inputs of ≥100 ng. The 4150 TapeStation system (Agilent, USA) was used for the quantification of nucleic acids at several steps throughout the protocol. The PCR enrichment step used the NEBNext multiplex oligonucleotides for Illumina (96 unique dual-index primer pairs) (New England Biolabs). The library prep samples were diluted to a final concentration of 1 nM with nuclease-free H_2_O and pooled. For loading onto the iSeq 100, the library was further diluted to 50 pM with nuclease-free H_2_O. Sequencing was run in paired-end 2 × 150-bp format with dual (8-bp + 8-bp) indices.

Isolates 9A-1-1, 9A-2-7, 9A-3-9, and 9A-4-9 were sequenced using long-read technology on a MinION device (Oxford Nanopore Technologies, UK). Genomic DNA was extracted using a Fire Monkey high-molecular-weight DNA extraction kit (RevoluGen). Library prep was carried out according to the manufacturer’s protocol (Oxford Nanopore Technologies), using the EXP-NBD104 native barcoding kit and the SQK-LSK109 ligation sequencing kit. Sequencing was carried out using a FLOW-MIN106 R9.4.1 flow cell (Oxford Nanopore Technologies) on a MinION Mk1B sequencer, running for 72 h.

### Bioinformatics.

After the completion of short-read sequencing, the BCL files were demultiplexed and converted to fastq using Illumina bcl-convert v3.9.3. Adapters were trimmed using Trimmomatic.

Base calling of the long reads was carried out with MinKNOW v20.06.4 software using the Guppy v4.0.9 algorithm. The long-read sequences were filtered using Filtlong (https://github.com/rrwick/Filtlong) with a minimum length threshold of 1,000 bp, keeping 90% of the best reads up to a total of 500 Mbp.

The filtered long reads were assembled using Flye v2.8.3-b1695 (31), which was found to outperform other long-read assemblers in benchmarking tests for bacterial whole-genome sequencing (WGS) (32). The Flye assembly used the following parameters: plasmids, trestle. The draft assembly, produced using Flye, was first checked for quality and visualized using Bandage (33). It was then polished with long reads using Medaka v1.5.0 (https://github.com/nanoporetech/medaka), polished with short reads using Polypolish v0.5.0 (34), and further polished with short reads using POLCA (https://github.com/Alekseyzimin/Masurca#Polca). Once polished with both short and long reads, the final assembly was annotated using RAST (35), with any unannotated or poorly annotated genes of interest checked using ISfinder (36), ResFinder (29), and BLAST (37). The assemblies were visualized using Bandage, and the annotated assemblies were visualized using SnapGene v3.3.4. The DNA sequences from Sanger sequencing were also visualized and analyzed using SnapGene v3.3.4.

### Consensus motifs for IS and Tn insertion sites.

The insertion sites for both IS*Apl1* and Tn*7511* throughout the 4 strains were analyzed to detect consensus within the insertion sites. The assembled genome was annotated with inverted repeats (IR), both left (IRL) and right (IRR), for both IS*Apl1* and Tn*7511* using ISfinder. The 30 bp upstream and downstream of the inverted repeats were collected and aligned to make a consensus motif using WebLogo (38).

### PCR to identify the putative (IS*Apl1*)_2_ circular intermediate.

PCR was carried out as described previously using transposant HMW genomic DNA as a template and the *mcr-1* outward primers from Table S1.

### Data availability.

The novel transposon-containing *mcr-1* was assigned the number Tn*7511* in the Transposon Registry (11). The WGS data from this study were deposited at the National Center for Biotechnology Information (NCBI) under BioSample accession numbers SAMN30184369 to SAMN30184372 and BioProject accession number PRJNA867121.
