# Supplementary material for: Intracellular Transposition of Mobile Genetic Elements Associated with the Colistin Resistance Gene mcr-1
Source: Microbiol Spectr. 2022 Dec 13;11(1):e03278-22. doi: 10.1128/spectrum.03278-22 (PMC9927407; doi:10.1128/spectrum.03278-22)

Supplementary Material

Supplementary Table 1: Primers used in PCR and primer walking within this study. Forward direction means 5” – 3” on sense top strand, whereas reverse direction means 5”-3” on the bottom strand. TET; Tetracycline.

Application	Primer Name	Target DNA Template	Direction	Primer Sequence (5’ – 3’)	Annealing Temp	Extension Time
PCR	*mcr-1*	pMCR-E2899	Forward	GCCTGTACGAATGTGCGAAG	53°C	15 sec
			Reverse	ATTCCGCATGGGTCATCGAG		
	cI-tetA	pBACpAK	Forward	CAGCCAGCAGAGAATTAAGG	57°C	MyTaq:<br>30 sec<br>(5 min after<br>TET exposure)<br><br>Q5:<br>5 – 10 min after TET exposure
			Reverse	GCAAGACTGGCATGATAAGG		
	tetA	pBACpAK	Forward	ACCACCTCAGCTTCTCAACG	51°C	30 sec
			Reverse	GTAAAGCGATCCCACCACCA		
Primer Walking (Sanger Sequencing)	*cI* repressor Middle Fwd	pBACpAK (Primer walking)	Forward	ATAAAGCACCAACGCCTGAC	N/A	N/A
			Reverse	N/A		
	IS*Apl1* - post IRL - Fwd (Tn*7511*)	pBACpAK (Primer walking)	Forward	GCTGAAACGACACGCTCTAG	N/A	N/A
			Reverse	N/A		
PCR	*mcr-1* outward primer	Transposant genomic DNA	Forward	GCAACACTCGCCACAAGAAC	50°C	32 sec
			Reverse	CACCGCGGACAAAGTCAAAG		

Supplementary Figure 1: Consensus sequences of IS*Apl1* and Tn*7511* insertion sites. The bases 30bp upstream and 30bp downstream of the Inverted Repeats (IR) flanking the IS and Tn element insertion sites were collected and aligned to make a consensus graph. (**A**) Alignment of insertion sites where IS*Apl1* inserted in the 5'-IRR-IRL-3' orientation (*n* = 18) and insertion sites where IS*Apl1* inserted in the 5'-IRL-IRR-3' orientation (*n* = 13). (**B**) Alignment of all insertion sites involving IS*Apl1* (*n* = 31), 18 sites were converted to their reverse complement prior to alignment (**C**) Alignment of target sites where Tn*7511* inserted (*n* = 2).

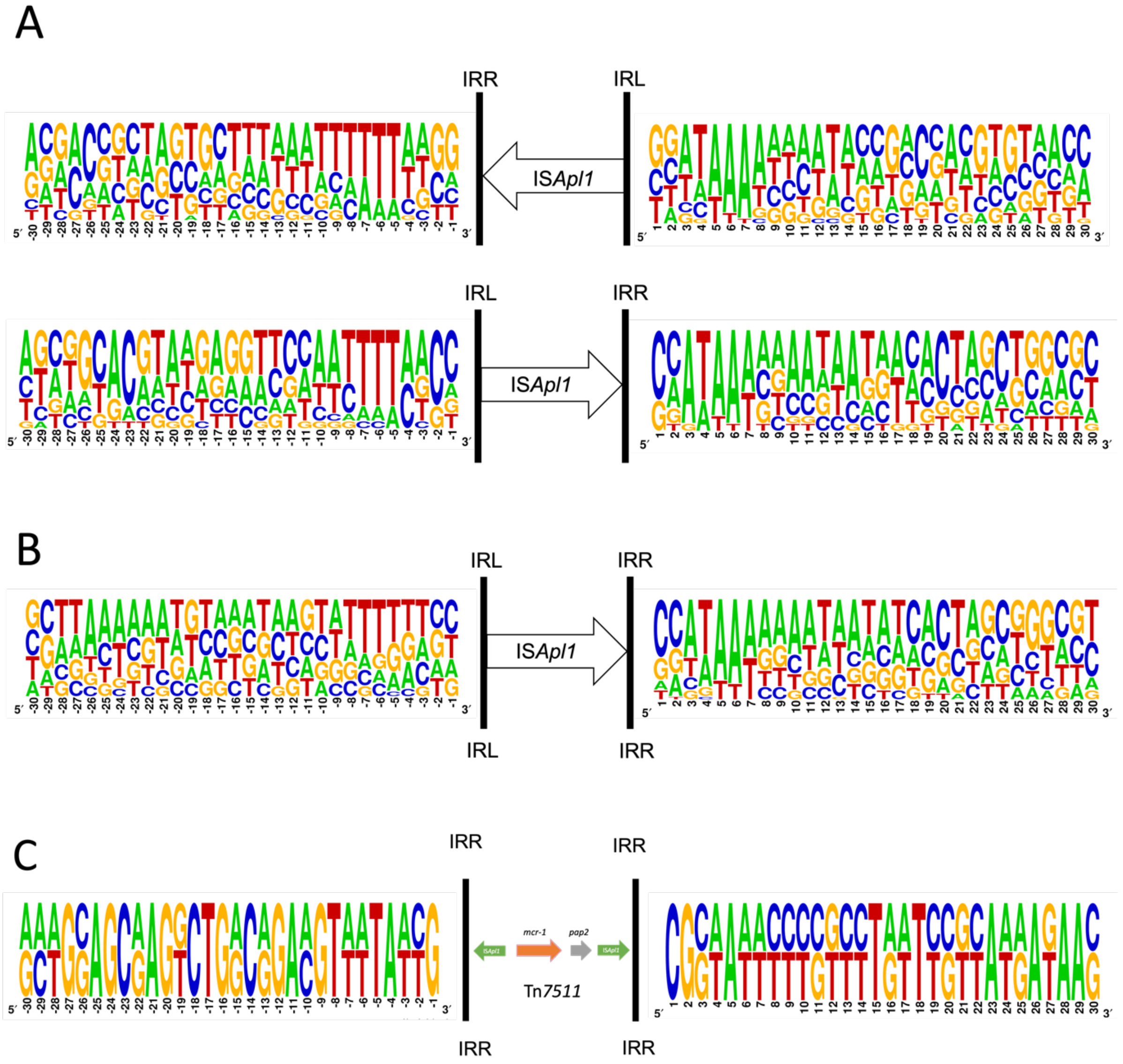

Supplementary Figure 2: Diagrammatic comparison of Tn*6330*, Tn*6390* and Tn*7511* transposons.

All three have the sequence IS*Apl1*-*mcr-1*-*pap2*-IS*Apl1* but the IS*Apl1* are in different orientations.

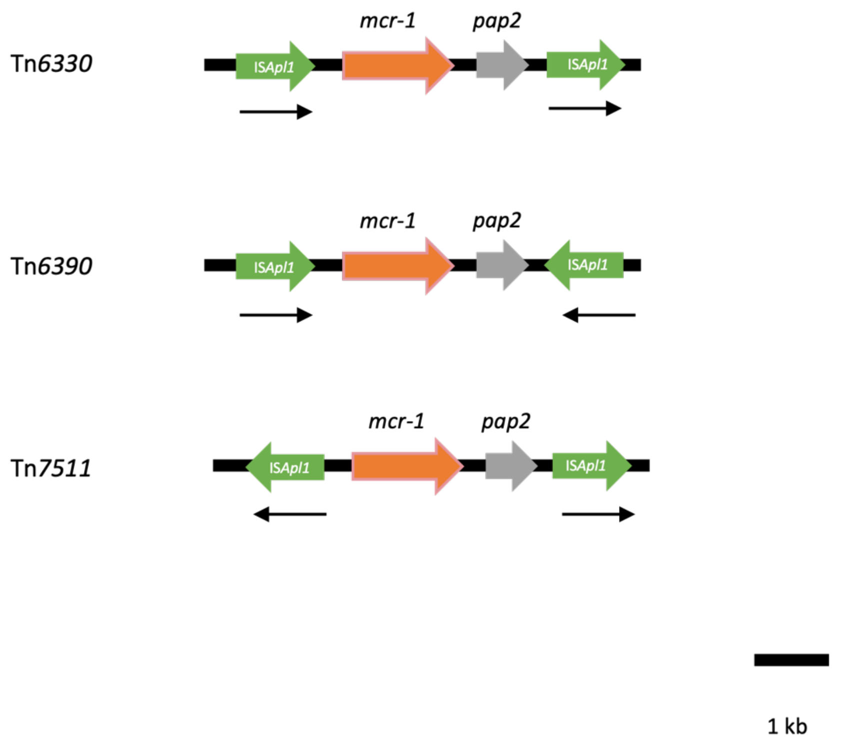

Supplementary Figure 3: Tn*7511* insertion site within 9A-1-1 chromosome aligned with the same genomic site in the other transposants. Visualised and aligned in Snapgene v3.3.4. The highlighted sequence shows the CG TSD flanking Tn*7511* within the 9A-1-1 chromosome.

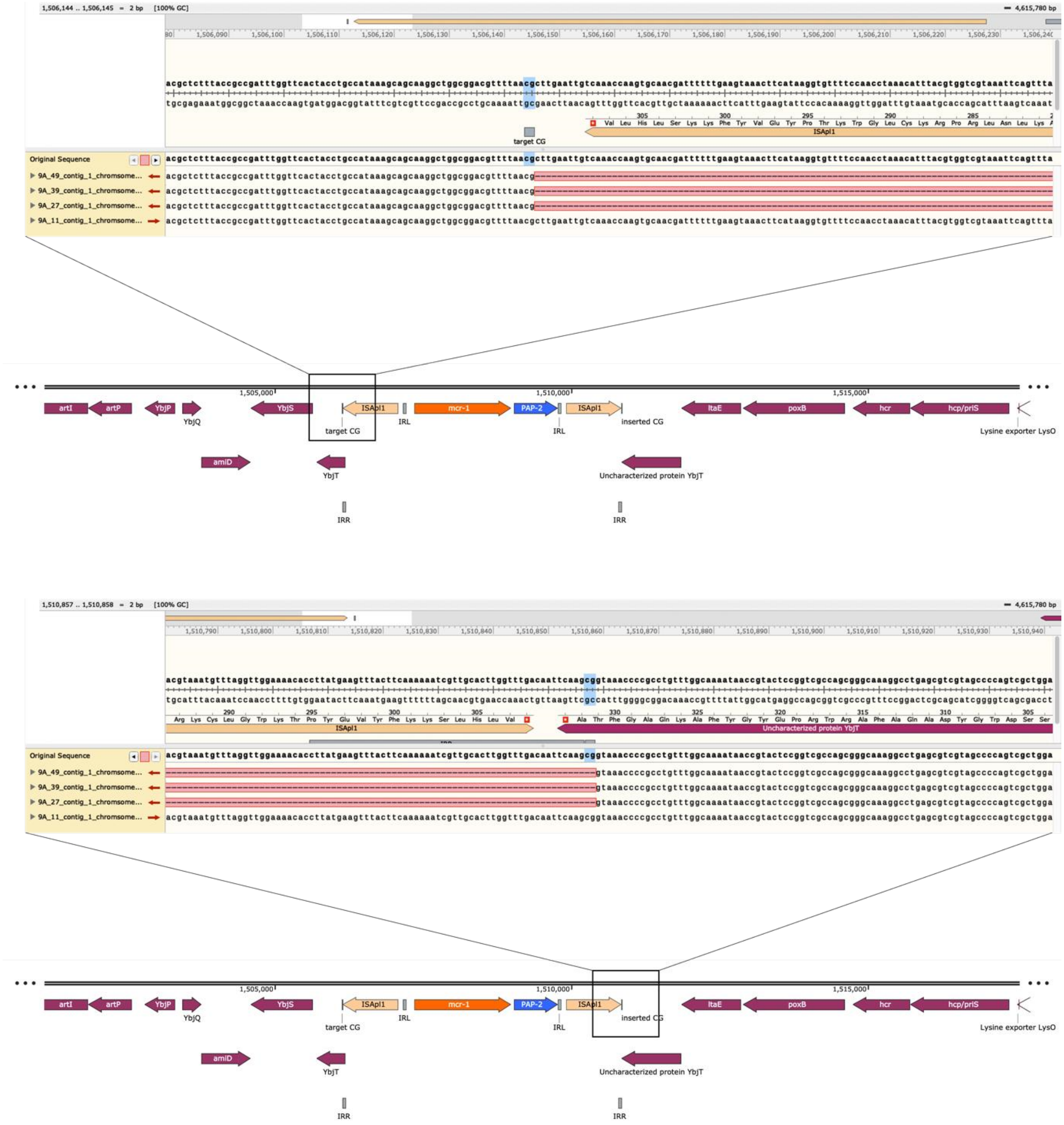

Supplement: Supplemental file 1 — Supplemental material. Download spectrum.03278-22-s0001.pdf, PDF file, 1.5 MB [file spectrum.03278-22-s0001.pdf]
